# Is response to IVIG in Chronic Idiopathic Urticaria related to presense of autoantibodies?

**DOI:** 10.1186/1710-1492-10-S1-A65

**Published:** 2014-03-03

**Authors:** Lana Rosenfield, Chrystyna Kalicinsky, Richard Warrington

**Affiliations:** 1 Department of Internal Medicine, University of Manitoba, Winnipeg, Manitoba, Canada; 2 Section of Allergy and Clinical Immunology ,Department of Internal Medicine, University of Manitoba, Winnipeg, Manitoba, Canada

## Background

Treatment of Chronic Idiopathic Urticaria (CIU) may be challenging, as patients can be refractory to various treatments. This may be due to an autoimmune mechanism in its pathogenesis. CIU has been shown to be associated with autoantibodies to IgE or IgE receptor and with thyroid autoimmunity [1,2]. Treatments targeting this autoimmune pathogenesis are now being used, such as intravenous immunoglobulin (IVIG). We reviewed patients with CIU treated with IVIG to see if response to treatment correlated with presence of autoimmunity (antibodies to IgE/IgE receptor or Thyroidperoxidase (TPO) antibodies).

## Methods

A retrospective chart review of patients seen in Allergy and Clinical Immunology clinics at Health Sciences Centre and Grace Hospital in Winnipeg was completed. Inclusion criteria included treatment with IVIG with a clinical diagnosis of CIU. There were a total of 21 patients identified. 13 patients were treated with protocol A, 2g/kg load split between 4 doses given within a two week period followed by maintenance of 0.5 g/kg. Protocol B was used for 8 patients consisting of monthly IVIG (60-80g). Autoimmune blood work was recorded for Histamine Release Assay (HRA) and TPO. HRA testing implies the presence of antibodies to IgE or IgE receptor. Patients' response to IVIG was recorded.

## Results

HRA was done on 15 /21 patients and 5 of these patients tested positive. 3/5 were able to maintain remission with continued IVIG treatment, one relapsed after discontinuing IVIG following remission and one was unresponsive to IVIG. Anti-TPO was tested in 11 patients of which 3 were positive. Of these three patients, one was responsive to IVIG, one relapsed after initially being responsive and one was unresponsive. Overall 13/21 patients were responsive to IVIG. Of those who were responsive to IVIG 6 were HRA negative and 3 HRA positive of the 9 tested, and 4 TPO negative and 1 TPO positive of the 5 tested for TPO. When comparing protocols, 7/13 patients who underwent protocol A responded without relapse compared to 6/8 treated with protocol B.

## Conclusion

IVIG can be effective in some patients with CIU who are unresponsive to antihistamine treatment. A greater number of patients who were responsive to IVIG had negative results for the autoimmune tests we conducted. Therefore, based on our data, the response to IVIG cannot be determined by the presence of positive HRA or TPO antibody.

